# Influencing factors of neural tube malformation: a systematic review and meta-analysis

**DOI:** 10.4314/ahs.v25i1.45

**Published:** 2025-03

**Authors:** Xiangling Wu, Xin Bian, Qianying Zheng, Ye Gu, Yuanyuan Wang, Tianping Bao, Weina Zhou

**Affiliations:** Department 2 of Ultrasonography, Baoding NO.1 Central Hospital, Baoding 071000, China

**Keywords:** Neural tube malformation, influencing factors, meta-analysis

## Abstract

**Background:**

Neural tube malformation is a common congenital malformation and its influencing factors were still unclear. This paper aims to explore the main influencing factors of neural tube malformation, and provide reference for the primary prevention of neural tube malformation.

**Methodology:**

Case-control literatures on the influencing factors of neural tube malformation from 1990 to 2021 were searched from Chinese and English websites. The quality of the included literatures was evaluated according to Newcastle-Ottawa Scale (NOS) scale and data were extracted. Meta-analysis was performed on the data using funnel plot and Egger's est evaluated publication bias, and sensitivity analysis was performed by eliminating individual studies one by one.

**Results:**

A total of 49 case-control studies were included. Meta-analysis showed that the main influencing factors of neural tube malformation were folic acid (odds ratio (OR)OR=0.31, 95%CI: 0.20-0.47), fever (OR=3.02, 95%CI: 2.38-3.83), obesity (OR=1.76, 95%CI: 1.39-2.21), passive smoking (OR=1.91, 95%CI: 1.52-2.40). Antiepileptic drugs (OR=6.10, 95%CI: 2.58-14.43); Heavy metals (Zinc: OR=2.37, 95%CI: 1.06-5.30, mercury: OR= 4.61, 95%CI: 2.85-7.47).

**Conclusion:**

Prenatal supplementation with folic acid and zinc has been shown to reduce the risks of neural tube defects. It is recommended that women of childbearing age take folic acid and zinc supplements before and during pregnancy. Other factors such as fever, obesity, passive smoking, antiepileptic drugs, and mercury exposure have been associated with an increased incidence of neural tube abnormalities. Neurological tube abnormalities can be reduced by folic acid and zinc, which act as protective factors. The incidence of neural tube abnormalities is increased by fever, obesity, passive smoking, antiepileptic drugs, and mercury.

## Introduction

Neural tube defects (NTDs) are severe congenital defects of the central nervous system that occur due to the failure of neural tube closure during embryonic development. Spina bifida and anencephaly are the two most common forms of NTDs. NTDs are among the most common birth defects in the world, with a global incidence of 0.5-10/%^1^. Each year, approximately 300,000 children are born with NTDs worldwide, making it the third leading congenital disease after congenital heart disease and Down syndrome^2^.

In China, the incidence of NTDs is about 13.9 per thousand, accounting for 25-35% of birth defects^2^. NTDs can result in high rates of fetal death in utero and postnatal disability, which can impose a significant burden on society, families, and individuals.

Recent epidemiological studies have shown that NTDs are mainly the result of genetic and environmental factors, with the latter being a research hotspot. Environmental factors such as folic acid, fever, obesity, passive smoking, antiepileptic drugs, and heavy metals may contribute to the development of NTDs^1^,^2^. Although there are many studies on the factors influencing NTDs, no systematic analysis has been conducted to study them.

Therefore, the present study aims to systematically analyze the factors influencing NTDs and provide new insights into the influence of different factors, such as environmental, genetic, or socio-demographic factors. The study aims to explore the main influencing factors of NTDs and provide references for the primary prevention of NTDs. Neural tube defects (NTDs) are severe congenital defects of the central nervous system caused by the failure of neural tube closure during embryonic development. The two mostommon forms are spinal bifida and anencephaly. Neural tube malformation is one of the most common birth defects in the world, with an incidence of 0.5-10%. Every year, about 300,000 children are born with neural tube malformations worldwide. It is the third leading congenital disease after congenital heart disease and Down syndrome. The incidence of neural tube malformations in China is about 13.9 per thousand^1^, accounting for 25-35% of birth defects. Its complication is the high rate of fetal death in utero and postnatal disability, which brings serious burden to society, families and individuals. In recent decades, epidemiological studies have shown that neural tube malformation is mainly the result of genetic factors and environmental factors, especially the influence of environmental factors has become one of the research hotspots, involving folic acid^2^, fever, obesity, passive smoking, antiepileptic drugs and heavy metals. A search of the relevant literature revealed that there are many studies on the factors influencing neural tube malformation, but no systematic analysis has been conducted to study them. Therefore, the study focuses on systematic analysis by studying the factors influencing neural tube malformation to further provide new insights into the influence of different factors on neural tube malformation, such as the role of environmental factors, genetic factors, or socio-demographic factors.

The purpose of the present study was to explore the main influencing factors of neural tube malformation, and provide reference for the primary prevention of neural tube malformation.

## Methods

### Literature retrieval strategy

The literatures of case-control studies on the influencing factors of neural tube malformations published by English databases PubMed(https://pubmed.ncbi.nlm.nih.gov/), Web of Science(https://www.webofscience.com/) and Chinese databases China national knowledge infrastructure (CNKI) (https://www.cnki.net/) and Wanfang(http://www.wanfangdata.com.cn/) were searched by computer. The search period is from 1990 to 2021. The English and Chinese keywords were searched: neural tube defects, case-control study, Folic acid, fever, obesity, passive smoking, antiepileptic drugs and heavy metals.

### Literature inclusion criteria

(1) The study must be a case-control study investigating the influencing factors of fetal neural tube malformations, published in either Chinese or English language between 1990 and 2021A case-control study on the influence factors of fetal neural tube malformations published in China and abroad from 1990 to 2021.
(2) The exposure factors must include folic acid, fever, obesity, passive smoking, antiepileptic drugs, and heavy metalsExposure factors included folic acid, fever, obesity, passive smoking, antiepileptic drugs and heavy metals.
(3) The outcome indicators must be odds ratio (OR) values or 95% confidencentervals (95%CI). Outcome indicators were odds ratio (OR) value or 95% confidence interval (95%CI).
(4) The case group must consist of mothers with neural tube malformations, while the control group must consist of normal mothers. Mothers of neural tube malformations were in the case group and normal mothers were in the control group.

### Literature exclusion criteria

(1) The literature cannot be a review, report, or individual case report. Literature type: review, report or individual case report;
(2) The research methods cannot be non-case-control study or cohort study, and the OR value or 95%CI cannot be obtained from the articleThe research methods were non-case-control study and cohort study, and the OR value or 95%CI could not be obtained in the article;
(3) The amount of data in the article must not be too small, or there must not be any data errorsThe amount of data ithe article was too small or there were data errors.

### Data extraction and literature quality evaluation

Literature screening, data extraction and literature quality assessment were all carried out by 2 researchers.

The quality of the included studies was evaluated using the Newcastle-Ottawa Scale (NOS), which required a literature score of ≥6. The literature that met the inclusion criteria was thoroughly read and information from the literature was extracted, including: first author, time of publication, country of study, study name, study type, sample size, number of case groups, number of control groups, OR or 95% CI.

### Statistical analysis

Meta-analysis was performed on the literature data collected by Stata 14.0 software. The relationship between folic acid, fever, obesity, passive smoking, antiepileptic drugs, heavy metals and neural tube malformation was evaluated by odds ratio (OR) and 95%CI. Heterogeneity was tested by Q test and I2. If P≥0.10 and I2≤50%, indicating that there was no statistical significance in heterogeneity between studies, fixed effect model was used for calculation, otherwise, random effect model was used for calculation. Publication bias was evaluated using funnel plot symmetry and Egger's test. Sensitivity analysis was performed by eliminating individual studies one by one.

## Results

### Literature screening results

**Figure 1:**
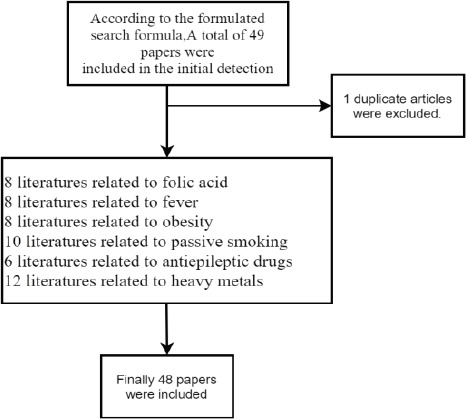
The inclusion of the enrolled studies

According to inclusion and exclusion criteria, a total of 49 literatures were included 3-51, including 42 English literatures written in English and 7 Chinese literatures written in Chinese. There were 8 literatures related to folic acid, 8 literatures related to fever, 8 literatures related to obesity, 10 literatures related to passive smoking, 6 literatures related to antiepileptic drugs, and 12 literatures related to heavy metals (including 7 literatures related to mercury and 6 literatures related to zinc), among which duplicate literatures were accumulated by one(Figure 1.). The NOS scores of all references were ≥6.

### Meta-analysis results

**Figure 2:**
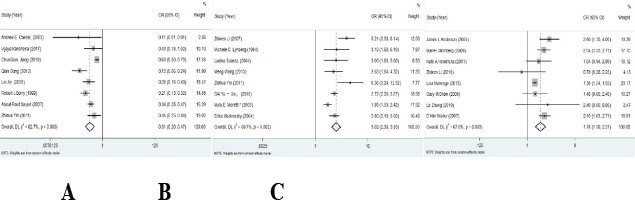

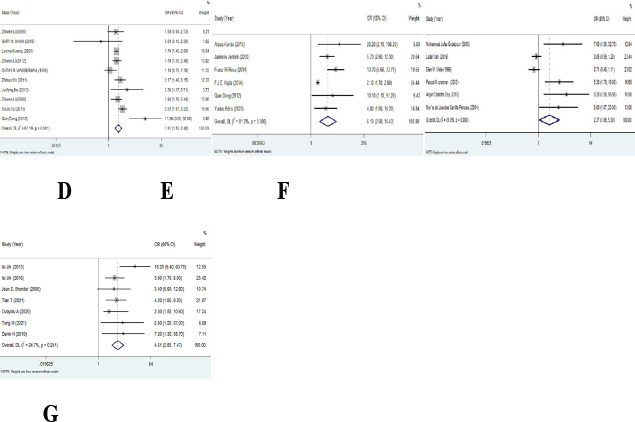
(A) Meta-analysis of folic acid and the risk of neural tube malformation forest map; (B) Meta-analysis of fever and the risk of neural tube malformations; (C) Meta-analysis of obesity and neural tube malformation risk forest map; (D) Meta-analysis of passive smoking and the risk of neural tube malformations; (E) Meta-analysis of antiepileptic drugs and the risk of neural tube malformations; (F) Meta-analysis of zinc and the risk of neural tube malformations; (G) Meta-analysis of mercury and the risk of neural tube malformation forest map

According to literature reports, folic acid, fever, obesity, passive smoking, antiepileptic drugs and heavy metal factors were selected as research objects to analyze their relationship with neural tube malformation. Heterogeneity test results showed that there was heterogeneity among folic acid, fever, obesity, passive smoking, antiepileptic drugs and zinc, and the effect size was combined by random effect model. There was no heterogeneity among studies of mercury in heavy metals, and the fixed effect model was used to combine the effect size. Meta-results showed that folic acid and zinc supplementation were protective factors for neural tube abnormalities, while fever, obesity, passive smoking, antiepileptic drug use and heavy metal mercury were risk factors for neural tube abnormalities in pregnant women (Table 1, Figure 2).

**Table 1:** Heterogeneity test and meta-analysis results of influencing factors of neural tube malformation

		Heterogeneity test			OR and 95%CI were combined	Combined P value
Influencing factors	Included literature	P	I^2^(%)	Effect model		
Folic acid	8(2-9)	<0.001	82.7	Random effect model	0.31(0.20,0.47)	< 0.001
pyrexia	8(9-16)	0.002	69.7	Random effect model	3.02(2.38,3.83)	< 0.001
fatness	8(17-24)	0.003	67.5	Random effect model	1.76(1.39,2.21)	< 0.001
Passive smoking	10(7,9,25-32)	0.001	67.1	Random effect model	1.91(1.52,2.40)	< 0.001
antiepileptic	6(7,33-37)	<0.001	81.2	Random effect model	6.10(2.58,14.43)	< 0.001
Heavy zincs	6(38-43)	<0.001	81.8	Random effect model	2.37(1.06,5.30)	< 0.001
metal mercuric	7 (44-50)	0.241	24.7	Fixed effect model	4.61(2.85,7.47)	< 0.001

### Sensitivity analysis and publication bias

**Figure 3:**
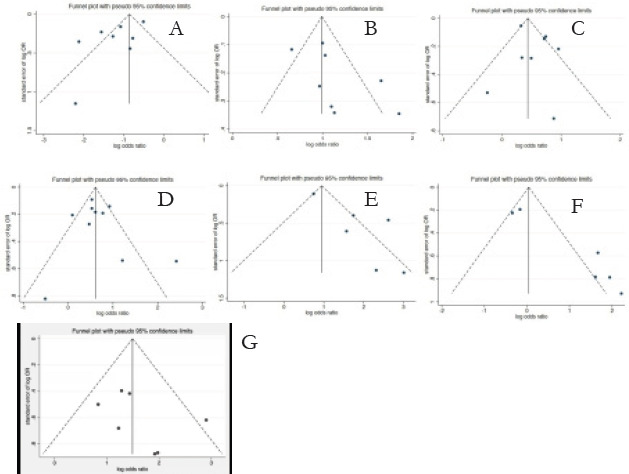
(A) Funnel plot of meta-analysis of folic acid and the risk of neural tube malformation; (B) Funnel plot of meta-analysis of fever and the risk of neural tube malformation; (C) Funnel plot of meta-analysis of obesity and the risk of neural tube malformation; (D) Funnel plot of meta-analysis of passive smoking and the risk of neural tube malformation; (E) Meta-analysis of antiepileptic drugs and the risk of neural tube malformation; (F) Funnel plot of meta-analysis of zinc and the risk of neural tube malformation; (G) Funnel plot of a meta-analysis of mercury and the risk of neural tube malformation;

The sensitivity analysis was conducted by eliminating individual studies one by one, and the results did not change significantly, indicating that the results of this study are basically reliable. Literature publication bias was evaluated by funnel plot and Egger's test, and funnel plot E and F (antiepileptic drugs and zinc) were asymmetric, while others were basically symmetric (Figure 3). In order to ensure the reliability of the result, continue to undertake Egger's test, the result suggested that antiepileptic drug exists to publish bias (P=0.02), the P value of a few other influencing factors all >0.05. Our meta-analysis found a significant association between the following risk factors and NTDs: folic acid, fever, obesity, passive smoking, and exposure to heavy metals zinc and mercury. The results of our meta-analysis were statistically stable and reliableThe Meta analysis conclusion of folic acid, fever, obesity, passive smoking, heavy metal zinc and mercury these risk factors was reliable. Publication bias of antiepileptic drugs was evaluated by clip-and-supplement method, and the results were basically consistent before and after clip-and-supplement, suggesting that meta-analysis conclusions of antiepileptic drugs were stable and reliable.

## Discussion

Primary prevention refers to taking measures aimed at the cause or risk factors to reduce harmful exposure levels and prevent the occurrence of diseases before they occur. To identify the risk factors for neural tube malformations and reduce their incidence at the primary prevention level, we conducted an analysis of the factors that may influence their occurrence. We analyzed the factors that may influence the occurrence of neural tube malformations, aiming to find the factors that can control the occurrence of neural tube malformations at the level of primary prevention and reduce the incidence of neural tube malformations and the burden on society, family and individuals.

### Folic acid

Folic acid is a widely studied teratogen that has been shown to be protective in preventing neural tube abnormalities. The use of folic acid supplements before pregnancy has been recommended as a public health policy to reduce the incidence of neural tube malformations. Numerous experimental and observational clinical studies over the past half century have demonstrated its effectiveness in reducing the risk of neural tube malformations by 50-70%^52^. Folic acid supplementation can significantly reduce the incidence of neural tube malformations or perinatal or neonatal mortality due to neural tube malformations.

One study found that folic acid is a protective factor for neural tube malformation, and folic acid supplementation can effectively prevent neural tube malformation through meta-analysis. The recommended intake of folic acid is 4 mg per day for women at high risk of neural tube abnormalities, and 0.4 mg per day for other women. In addition to pre-pregnancy folic acid supplementation, some countries have introduced mandatory folic acid fortification of foods to reach women with unplanned pregnancies and women in poor areas.

After folic acid fortification in South Carolina, the rate of neural tube defects fell by 58% in 2009 compared with 1992^53^. The mechanism of folic acid preventing neural tube malformation is less well studied than folic acid in the field of epidemiology, but ongoing research into the mechanisms by which folic acid prevents neural tube malformations in animal models may provide further insight.

However, one issue with the protective effects of folic acid is the timing of its use. The development and closure of the neural tube typically occurs within 28 days after the last menstruation of a pregnant woman, but many women do not realize they are pregnant until about 40 days after their last menstrual period^54^. By this time, the differentiation of the neural tube may already be completed, making the protective effect of folic acid uncertain. Ongoing research is needed to determine the efficacy of folic acid when taken after the critical period of neural tube development. Folic acid supplements before pregnancy have been studied for the prevention of neural tube abnormalities since the 1980s. Folic acid has been shown to be protective in 50-70% of neural tube malformations in numerous experimental and observational clinical studies over the past half century, and folic acid supplementation can significantly reduce the incidence of neural tube malformations or perinatal or neonatal mortality due to neural tube malformations^52^. In this study, through meta-analysis, it was found that folic acid is a protective factor for neural tube malformation, and folic acid supplementation can effectively prevent neural tube malformation. Folic acid supplementation is now a public health policy. The recommended intake of folic acid is 4 mg per day for women at high risk of neural tube abnormalities, and 0.4 mg per day for other women. In addition to pre-pregnancy folic acid supplementation, some countries have introduced mandatory folic acid fortification foods to reach women with unplanned pregnancies and women in poor areas. After folic acid fortification in South Carolina, the rate of neural tube defects fell by 58% in 2009 compared with 1992^53^.

The mechanism of folic acid preventing neural tube malformation is far less well studied than folic acid in the field of epidemiology. Currently, there are two hypotheses: one is that folic acid enhances cell proliferation; the other is that methylation of folic acid involves epigenetic regulation^54^. Ongoing research into the mechanisms by which folic acid prevents neural tube malformations in animal models may resolve this confusion in the coming years.

But there is a problem with the protective effects of folic acid. The development and closure of the neural tube is usually completed within 28 days after the last menstruation of a pregnant woman, while pregnant women generally do not realize pregnancy until about 40 days after menopause. Many women have completed the differentiation of the neural tube before they realize pregnancy, but some pregnant women start to take folic acid at this time. What is the protective effect of folic acid on neural tube abnormalities at this time?

### Heating

Regarding heating as a teratogen, we have included evidence from epidemiological and animal studies that suggests that high temperature is a teratogenic agent. Our study found that maternal exposure to common cold, flu, and febrile illness early in pregnancy was associated with an increased risk of neural tube abnormalities. Specifically, mothers who had fever or flu during pregnancy had a four-fold increased risk of neural tube abnormalities in their fetuses^55^. The risk of anencephaly was also slightly higher in febrile pregnant women than in spina bifida.

We have also included evidence that suggests that the use of hot tubs alone can increase the incidence of neural tube malformation in pregnant women exposed to heat, and that the use of bathtubs, saunas, electric blankets, and other exogenous heating environments may also increase the risk^56^.

While it is difficult to know whether this association is due to febrile disease or fever itself, most studies report fever as a risk factor for neural tube abnormalities, which is consistent with our findings^57^. Therefore, we suggest that pregnant women with fever should be vigilant, and that pregnant women should avoid intense exposure to high temperatures.

The study found an association between maternal exposure to common cold, flu, and febrile illness early in pregnancy and an increased risk of neural tube abnormalities, with mothers who had fever or flu during pregnancy having a four-fold increased risk of neural tube abnormalities in their fetuses^55^. The results also showed that the risk of anencephaly was slightly higher in febrile pregnant women than in spina bifida^56^. Our results suggest that maternal hyperthermia or febrile illness are risk factors for neural tube abnormalities. Data froanimal studies suggest that high temperature is itself a teratogenic agent, while data from epidemiological studies cannot fully unravel the potential teratogenic mechanisms of temperature increases or diseases that cause them^57^. Evidence showed that by using hot tubs alone, pregnant women exposed to heat were 3 times more likely to develop a pregnancy with neural tube abnormalities. In other words, the use of bathtub, sauna, electric blanket and other exogenous heating environment may also increase the incidence of neural tube malformation. Therefore, as epidemiological and experimental data accumulate, we suggest that pregnant women with fever should be vigilant, and that pregnant women should avoid intense exposure to high temperatures. Although it is difficult to know whether this association is due to febrile disease or fever itself, most studies report fever as a risk factor for neural tube abnormalities, which is consistent with our findings.

### Obesity

In this study, meta-analysis found that obesity in pregnant women was a risk factor for neural tube malformation. Other reported risks associated with maternal obesity include heart malformations, stillbirth, macrosomia, meconium inhalation and adolescent obesity. Compared with women with a normal body mass index (BMI), the risk of spina bifida was approximately 2 times higher in the offspring of obese women, and the risk of neural tube malformation was 1.5 to 3.5 times higher in the offspring of obese women than in women with a lower BMI^58^. A systematic review and meta-analysis recently published in the Journal of the American Medical Association showed that every 5-unit increase in maternal bmi was associated with a further increase in fetal and neonatal deaths and stillbirths. Therefore, women who plan to become pregnant should combine with weight management guidelines and control their BMI not to exceed 28-30 kg/m2^59^. Obesity is a risk factor for neural tube abnormalities, and its biological mechanism has not been clarified, just as the correlation between obesity and metabolic abnormalities, insulin resistance and hyperglycemia has not been clarified.

At present, there is no large-scale data report on the relationship between obesity anneural tube malformations due to different reasons. Either the sample size of the study is small, or it is limited to the relationship between hyperglycemia and metabolic abnormalities and neural tube malformations. For example, studies have shown that the offspring of pregnant women with hyperglycemia are 2-10 times more likely to suffer from neural tube malformations, and body mass index is not included in the study as one of the grouping factors. We expect to be able to collect a sufficient number of large sample studies to analyze the influence of obesity on neural tube malformation and whether the pathogenesis is the same.

### Passive smoking

The harm of passive smoking to pregnant women is huge, and our meta-analysis shows that passive smoking is a risk factor for neural tube abnormalities. Many of the 7,000 chemicals in smoke easily cross the placenta and have direct adverse effects on subsequent pregnancy outcomes, such as intrauterine growth restriction, congenital heart disease, neural tube malformations, etc. The relationship between maternal passive smoking during pregnancy and some adverse pregnancy outcomes has been well studied^60^,^61^. The mechanism by which maternal passive smoking may lead to neural tube malformations remains to be determined. Some studies hypothesize that passive smoking may reduce serum folic acid levels and increase homocysteine levels^62^, which is a sensitive indicator of folic acid deficiency. In animal models, teratogenicity of cigarette smoke components has also been implicated in other mechanisms, including nicotine, carbon monoxide, and cadmium. Therefore, as part of pre-pregnancy education, fathers should be advised to quit smoking to eliminate their risk associated with birth defects. Smoking cessation by fathers during early pregnancy is not effective in reducing the risk of neural tube abnormalities, and therefore smoking cessation during early pregnancy should be advocated, the study reported.

### Antiepileptic drugs

Several commonly used clinical antiepileptic drugs (AEDs) include sodium valproate, carbamazepine, phenytoin sodium, lamotriazine, etc., which have their respective advantages in the prevention of partial epileptic seizures, but sodium valproate (VPA) is widely regarded as the most effective treatment for the control of primary epileptic seizures. More recently, its use has expanded as a prophylactic for controlling migraines and as a first-line drug for some types of schizophrenia. It has been used to treat epilepsy for more than 30 years, and there is growing evidence that valproate is an important human teratogenic agent. We found that antiepileptic drugs were a risk factor for neural tube malformation through meta-analysis.

In the 1980s, pregnant women receiving valproate monotherapy during the first trimester of pregnancy were first reported to be associated with an increased risk of congenital spina bifida. Subsequent studies confirmed this increased risk and suggestd an increased risk for other major congenital malformations, with strong data suggesting that valproate exposure increased the risk of neural tube defects by 12%. In a population-based case-control study, we found a significant association between sodium valproate monotherapy in early pregnancy (compared with no antiepileptic therapy) and six conditions: atrial septal defect, cleft palate, hypospadias, polydactylia, craniosynostosis, and spina bifida. The risk of the first five conditions is 2-7 times that of a normal fetus, while the sixth condition is 12-16 times that of spina bifida. The risk increases with the dose. When we compared valproate exposure to other antiepileptic drug exposures, the risk for five of these six abnormalities remained significantly increased. Recently, the American Academy of Neurology recommended avoiding valproate use during pregnancy if possible. However, if valproic acid treatment is good at controlling seizures, it is difficult to change medications before or during pregnancy, so the risks of valproic acid use should be routinely considered when treating women with reproductive needs.

### Heavy Metals

Prenatal exposure to heavy metals mercury, lead, cadmium, and arsenic induced neural tube malformations in a variety of animal models. The association between heavy metals and neural tube abnormalities was supported by animal models. In our meta-analysis, zinc and mercury, heavy metals with a large sample size and frequent daily exposure, were selected as representatives. The results showed that zinc was a protective factor for neural tube malformations, zinc supplementation can reduce the incidence of neural tube malformations, and mercury was a risk factor for neural tube malformations. Heavy metals enter the mother's body mainly through respiratory system, skin contact and ingestion with food, alcohol, smoking or cosmetics. In the subsequent studies on the relationship between fetal neural tube malformations and heavy metals, mercury and mercury, as the most common heavy metals, have a large sample size and good representativeness. Lead, cadmium and other heavy metals may also be related to neural tube malformations, but the sample size of the study was small, which was not suitable for meta-analysis. Mothers of fetuses with neural tube defects have lower blood zinc concentrations than normal pregnant women. Zinc, a protective factor for neural tube defects, has been elevated in recent years to levels as important as folic acid. Zinc is a core component of more than 300 enzymes and proteins, and any factor that reduces plasma zinc may lead to functional zinc deficiency or teratogenic effects, especially during critical periods of organ development, such as neural tube development^40^. Mercury is a well-known neurotoxin and teratogenic agent^47^. Prenatal mercury exposure increases the risk of neural tube abnormalities. There are two possible mechanisms associated with mercury exposure and neural tube malformations. Te first mechanism is proapoptotic. Mercury promotes apoptosis, and excessive apoptosis can lead to neural tube malformations, such as insufficient cells in nerve fold fusion or disruption of continuity in the dorsal midline. Animal experiments have shown that mercury can induce apoptosis in a dose-dependent manner. The second mechanism is oxidative stress. Mercury is metabolized in the body to produce free radicals, which interfere with the function of glutathione peroxidase and other antioxidant enzymes, and induce lipid peroxidation and neurotoxicity. Long-term exposure to mercury increases lipid peroxidation and nitrite concentrations and reduces total antioxidant capacity.

## Limitations

The studies included in the review may not be representative of all studies conducted on the topic, since studies with null or negative findings may not be published as frequently. Additionally, all potential confounding factors may not have been accounted for in the studies included in the review, which may affect the accuracy of the results.

## Conclusion

The birth of children with neural tube malformation has brought huge economic and mental burden to society, families and individuals, which is an important content in the field of public health. We hope to advocate pregnant women to take folic acid and zinc supplements actively, effectively prevent and control fever, obesity, passive smoking, antiepileptic drugs and heavy metals, improve the efficiency of primary prevention of neural tube malformation and reduce the incidence of neural tube malformation by analyzing the influencing factors of neural tube malformation.
